# Editorial: New Insights Into Oxidative Stress and Inflammation in the Pathophysiology and Treatment of Cardiovascular Diseases

**DOI:** 10.3389/fmolb.2022.940465

**Published:** 2022-06-24

**Authors:** Matteo Becatti, Antonio Abbate, Claudia Fiorillo, Roberto Carnevale, Santosh Kumar

**Affiliations:** ^1^ Department of Experimental and Clinical Biomedical Sciences “Mario Serio”, University of Firenze, Firenze, Italy; ^2^ VCU Pauley Heart Center, Virginia Commonwealth University, Richmond, VA, United States; ^3^ Department of Medical-Surgical Sciences and Biotechnologies, Sapienza University of Rome, Rome, Italy; ^4^ Cardiovascular Division, Department of Medicine, and Abboud Cardiovascular Research Center, University of Iowa Carver College of Medicine, Iowa City, IA, United States

**Keywords:** oxidative stress, reactive oxygen species, inflammation, cardiovascular diseases, aging

Cardiovascular diseases (CVD), the leading cause of morbidity and mortality globally, represent a major concern for health, social, and economic services. More than four million Europeans die every year from CVD, primarily from coronary heart disease and stroke, accounting for 45% of all deaths (Movsisyan et al., 2020). In addition, SARS-CoV-2 infection has exacerbated the CVD burden by being strictly associated with heart failure, thrombosis, stroke, and pulmonary embolism (Regional Health–Europe, The Lancet Regional, 2021). Although novel pharmacological and technological treatments have improved survival, a multitude of patients live for many years with debilitating cardiovascular problems and low quality of life. Hence, a better understanding of the mechanisms underlying CVD is crucial for improved management or prevention. In the current Research Topic (RT), an overview of the new trends and achievements regarding the role of inflammation and oxidative stress in CVD is provided.

Clinical and experimental studies have already demonstrated a close relationship between inflammation and oxidative stress in CVDs. The antioxidant and immune-suppressant properties of the most successful cardiovascular drugs (e.g., statins, ACE inhibitors, and AT1R blockers) (Umebayashi et al., 2019; Mansouri et al., 2022) and the lowered cardiovascular mortality of anti-inflammatory therapy in autoimmune diseases such as psoriasis, rheumatoid arthritis (Roubille et al., 2015) and systemic lupus erythematosus (Kostopoulou et al., 2020) support a link between cardiovascular disease and chronic autoimmune diseases through inhibition of inflammation and oxidative stress (Emmi et al., 2019). This evidence has been further strengthened by the dramatic increase in cardiovascular events in COVID patients, where cytokine storm represents the main biological phenomenon.

The inflammatory biomarkers, in particular circulating C-reactive protein (CRP) and interleukin IL-6, which have been associated with vascular risk in humans, represent potential targets for CVD prevention (Ridker, 2016). Indeed, recent trials provided proof-of-concept for the inflammatory origin of CVD (Nidorf et al., 2020). In the Canakinumab Anti-inflammatory Thrombosis Outcome Study (CANTOS) a monoclonal antibody against IL-1β has been shown to lower the risk of recurrent vascular events among individuals with recent myocardial infarction (Ridker et al., 2017; Ridker et al., 2018). The Colchicine Cardiovascular Outcomes Trial (COLCOT) and the Low-Dose Colchicine-2 (LoDoCo2) trial further showed that the anti-inflammatory drug colchicine dramatically reduces the risk of recurrent vascular events in CAD patients (Opstal et al., 2020). Notably, neither the CANTOS nor the colchicine trials reduced mortality and both canakinumab and colchicine were associated with adverse effects including fatal infections (Tardif et al., 2019). Hence, alternative improved anti-inflammatory drugs are needed.

Over 20 years of experimental data have shown that anti-inflammatory strategies are effective in the prevention of CVD. Great success has been reached by targeting the interleukin-1β-interleukin-6 pathway. However, inter-individual Research Topic in drug response and a high rate of side effects highlight the need for a second generation of anti-inflammatory agents (Lutgens et al., 2019).

The close link between inflammation and redox balance is also supported by data on aggravated inflammatory phenotype in case of oxidative stress conditions -due to inadequate antioxidant defenses and/or reactive oxygen species (ROS) overproduction (Aimo et al., 2020). During inflammatory response, leukocytes and mast cells induce a “respiratory burst,” releasing ROS and inflammatory mediators -such as cytokines and chemokines-, which, in turn, promote further ROS release in the damaged area. Therefore, inflammatory pathways may represent effective targets in the treatment of CVD. In this RT, Villar-Fincheira et al. summarize the dual role of IL-6 in both innate and adaptative immune responses explaining the opposite effects of this protein during inflammation and exercise, with a special focus on the vascular system and vascular diseases.

CVD is a leading contributor to morbidity and mortality in the elderly and is closely correlated with age. Inflammation and oxidative stress in the elderly are not only associated with CVD but also with other age-related disorders, such as chronic obstructive pulmonary disease, chronic kidney disease, neurodegenerative diseases, and cancer (Liguori et al., 2018). More recently, it has been suggested that aging and CVD are strictly interconnected and may share common pathways. New data indicate that mitochondria within cardiomyocytes contribute to age-related increased ROS generation which is associated with aging-associated cardiac diseases. In particular, age-related alterations in patients were identified in mitochondrial-electron transport chain-complex-I (Rizvi et al., 2021).

Increased interest in the potential contribution of CVD in Alzheimer’s disease pathogenesis has arisen because several vascular risk factors (i.e., hypertension, metabolic syndrome, hypercholesterolemia, atherosclerosis, arterial stiffness) have been associated with this dementia syndrome (Ferrucci and Fabbri, 2018; Tavenier et al., 2020). In the current RT, Hendrickx et al. discuss the role of oxidative stress and chronic low-grade inflammation as the mechanistic convergence between CVD and Alzheimer’s disease highlighting the key role of arterial stiffness in Alzheimer’s disease progression. The authors underline the importance of an early diagnosis of inflammaging and arterial stiffness monitoring to prevent dementia progression.

Several data highlight the crucial roles of ketone bodies (β-hydroxybutyrate (β-OHB), acetoacetate, and acetone) in the pathophysiological progression of CVD (Selvaraj et al., 2020). In certain physiological states, such as fasting, starvation, and low-carbohydrate diets, ketone bodies become a significant source of overall energy and the substrate of mammalian metabolism in the extrahepatic tissues (Puchalska and Crawford, 2017). In addition, β-OHB can serve as an endogenous histone deacetylase (HDAC) inhibitor, which is associated with increased global histone acetylation to initiate the transcription of antioxidant genes in response to oxidative stress (Chriett et al., 2019). In light of the regulatory effects of β-OHB on oxidative stress and inflammation, the research progress of β-OHB in CVD has been discussed by Wei et al., exploiting the potentials of β-OHB in cardiovascular therapies. Indeed, the prevalence of metabolic disturbances such as metabolic syndrome and diabetes significantly increases with age and further contributes to CVD morbidity and mortality. The powerful interplay between nutritional and metabolic alterations and cardiovascular disorders is also underlined by Durante et al. in an interesting original article. CVD is the most prevalent cause of morbidity and mortality in type 2 diabetes mellitus (T2DM) patients and ischemic heart disease is one of the most common causes of death in T2DM patients. There is substantial evidence that exposure to excessive fructose intake has detrimental effects on multiple cardiometabolic risk factors. In an animal model of ischemia/reperfusion (I/R) injury, Durante showed that chronic feeding with a high fructose diet induced drastic metabolic derangements, which were paralleled by worsening in the outcomes of cardiac I/R injury, as demonstrated by drastic increases in infarct size and markers of fibrosis, inflammation and oxidative stress. Interestingly, these effects were prevented when fructose intake was replaced by its isomer D-tagatose, a sugar with a lower caloric value used as a low-calorie sweetener. This study demonstrates that D-tagatose represents an interesting sugar alternative when compared to its isomer fructose with a reduced deleterious impact not only on the metabolic profile but also on the related heart susceptibility to I/R injury.

Overall, this RT provides a comprehensive Research Topic on the role of oxidative stress and inflammation in CVD, providing a detailed view of the field, from basic principles to therapeutics.
